# Inhibition of the PCR by genomic DNA

**DOI:** 10.1371/journal.pone.0284538

**Published:** 2023-04-21

**Authors:** Sue Latham, Elizabeth Hughes, Bradley Budgen, Alexander Morley

**Affiliations:** College of Medicine and Public Health, Flinders University, Adelaide, Australia; University of Helsinki: Helsingin Yliopisto, FINLAND

## Abstract

**Aims:**

qPCR, is widely used for quantifying minimal residual disease (MRD) and is conventionally performed according to guidelines proposed by the EuroMRD consortium. However it often fails when quantifying MRD levels below 10^−4^. By contrast, HAT-PCR, a recent modification designed to minimise false-positive results, can quantify MRD down to 10^−6^.

**Methods:**

The factors leading to failure of conventional qPCR to quantify low levels of MRD were studied by analysing PCR reagents, protocol and primers and by testing for inhibition by adding primers to a plasmid amplification system. Complementary primers, ending in either G/C or A/T, were used to determine the effect of the 3’ end of a primer.

**Results:**

Inhibition of conventional PCR resulted from interaction of primers with genomic DNA leading to exponential amplification of nonspecific amplicons. It was observed with approximately half of the EuroMRD J primers tested. Inhibition by a primer was significantly related to primer Tm and G/C content and was absent when extension at the 3’ end was blocked. Nonspecificity and inhibition were decreased or abolished by increasing the annealing temperature and inhibition was decreased by increasing the concentration of polymerase. Primers terminating with G/C produced significantly more nonspecificity and inhibition than primers terminating with A/T. HAT-PCR produced minimal nonspecificity and no inhibition.

**Conclusions:**

Inhibition of the PCR may result from the presence of genomic DNA and resultant exponential amplification of nonspecific amplicons. Factors contributing to the phenomenon include suboptimal annealing temperature, suboptimal primer design, and suboptimal polymerase concentration. Optimisation of these factors, as in HAT-PCR, enables sensitive quantification of MRD. PCR assays are increasingly used for sensitive detection of other rare targets against a background of genomic DNA and such assays may benefit from similar improvement in PCR design.

## Introduction

Sensitive quantification of minimal residual disease (MRD), in lymphoid neoplasms is often performed by PCR, using a rearrangement of the immunoglobulin or T cell receptor gene as a target and a patient- or allele-specific oligonucleotide (ASO) as a primer. Since its initial description 3 decades ago ASO-qPCR has been widely used, particularly in Europe and publications by members of the EuroMRD consortium have provided guidelines on performance of the method and recommended sequences for generic reverse primers [1–3]. A candidate patient-specific forward primer, the sequence of which is unique and specific to the patient, is acceptable for use if it can quantify MRD down to a level of 10^−4^. Some primers can quantify below 10^−4^ but some fail [4]. Failure may sometimes be due to false positivity but often the cause is not clear.

HAT-PCR (**H**igh **A**/**T** PCR or **H**igh **A**nnealing **T**emperature PCR) is a recent modification of qPCR which involves improvement in primer design and amplification conditions in order to improve specificity and decrease the frequency of false-positive results in MRD assays [5]. It has a limit of detection of 10^−6^ when 20 μg of DNA are assayed^5^. The sensitivity of conventional qPCR, performed according to Euro MRD guidelines and using a patient-specific forward primer and a recommended reverse J primer, was studied following development of HAT-PCR. An individual primer pair usually detected levels of MRD down to 10^−4^ but frequently failed to detect lower levels.

The PCR can potentially amplify a single target to the point of detection [6] but amplification of the target can sometimes be inhibited either by an extrinsic agent which has co-purified with genomic DNA or by another intrinsic amplification reaction. Inhibition resulting from amplification of primer-dimers is common, and many technical modifications of PCR have been developed to minimise it [7, 8]. Amplification reactions of other off-target DNA sequences have also been observed [9] but such reactions have been poorly characterised, and their importance has been unclear.

The observed failure of conventional qPCR to quantify MRD below 10^−4^ proved to be due to interaction of primers with genomic DNA. Except for a report that fragmented genomic DNA [10] may inhibit the PCR, the role of genomic DNA in the PCR has aroused little interest. However, we felt it was important to analyse the phenomenon for 2 reasons. Firstly, its understanding and prevention could improve the sensitivity of quantification of MRD. Secondly, other PCR assays involve sensitive detection of a target in the presence of genomic DNA and may therefore be prone to inhibition. Analysing the mechanism of inhibition and its prevention could therefore be relevant to the design of many PCR assays.

## Methods

Samples of blood or marrow were obtained with informed consent from patients participating in study ALL06 (ACTRN12611000814976) of the Australasian Leukaemia and Lymphoma Group. DNA was extracted using the Qiagen Flexigene DNA kit and was quantified using the Invitrogen Qubit 3.0 Fluorometer.

All PCRs were quantitative PCRs performed using a Biorad CFX Connect Real-time PCR detection system and the results were analysed using Biorad CFX Manager 3.1 software.

Conventional qPCR was performed in accord with EuroMRD guidelines [2, 3]. The forward primers were patient-specific and the reverse consensus J primers were those recommended by EuroMRD for *IGH* and *TCRβ* rearrangements. The Tm of the forward primers was 62.5–67.5 and that of the reverse primers was 63.9–70.3. The constituents of the PCR were: KCl 50 mM, MgCl_2_ 3mM, dNTPs 200 mM, primers 300 nmol/l, probe 160 nmol/l, Taq polymerase 0.6U, Tris HCl 20 mM (ph 8.4), H_2_O in a volume of 25 μl. The amplification protocol was: 91°C for 3 minutes; 45 cycles of 94°C for 15sec and annealing at 60°C for 30sec.

HAT-PCR was performed using forward and reverse primers designed with a high Tm and with 1–4 A or T bases at the 3’ end^5^. The Tm of the forward primers was 69.0–71.0 and that of the reverse primers was 71.2–73.6. The constituents of the PCR were: KCl 50 mM, MgCl_2_ 5mM, dNTPs 300 mM, primers 400 nmol/l, hydrolysis probe 160 nmol/l, Taq polymerase 2U, Tris HCl 20 mM (ph 8.4), H_2_O in a volume of 25 μl. The amplification protocol was: 91°C for 3 minutes; 5 cycles of 97°C for 15sec and 72°C for 30sec; 5 cycles of 96°C for 15sec and 72°C for 30sec; 35 cycles of 94°C for 15sec and annealing at 72°C for 30sec.

Genomic DNA was DNA pooled from 5 non-leukemic individuals and was used at 0.5 μg / well, unless otherwise stated. A gene-specific hydrolysis probe was used to quantify specific amplification in all experiments and the non-specific fluorochrome, Syto82 at 0.5μM per well, was used to quantify nonspecific amplification. The number of leukemic targets / mass of DNA in diagnosis samples was determined by digital PCR. All PCRs contained 500 ng genomic DNA unless stated otherwise.

Plasmid 105–16 was constructed to contain a unique rearranged IGH sequence cloned into plasmid pGEM-T (Promega, Madison, WI). Sequencing showed that the plasmid contained a single target sequence, and the number of target sequences / mass of plasmid DNA was determined by digital PCR. Amplification of the plasmid target sequence used a specific forward primer and a generic *IGH* J6 primer, each at a concentration of 400 nmol/l. Primers were tested for inhibition by adding them to the plasmid amplification reaction and observing the effect on amplification of the plasmid target by the plasmid primers.

The following experimental designs were used to study inhibition:

- qPCRs containing decreasing masses of target DNA were performed, with or without added genomic DNA, and the Ct values were recorded. Unless otherwise stated, a Ct result was the mean of results from 3 wells.
- a measure of the degree of inhibition produced by a primer pair or an individual primer in the presence of genomic DNA was derived as follows and termed the inhibition index. qPCRs containing various masses of DNA were performed and the results scored as positive or negative. Analogous to the determination of the number of stem cells in a heterogeneous mixture of cells, the target sequences were regarded as being either capable or incapable of amplifying to produce a probe signal. Using limiting dilution analysis with Poisson statistics [11], the mass (m) of DNA containing one signal-producing sequence was then calculated either for wells containing DNA (m^+^) or not containing DNA (m^**-**^). The inhibition index was the ratio of m^**-**^/m^+-^. An example is shown in Table 2 in S1 Data.
- production of nonspecific DNA amplicons by a single test primer acting in the absence of its target was quantified by determining the Ct of a qPCR containing 900 nmol/l test primer, 500 ng genomic DNA and 0.5 μmol/L fluorochrome Syto82. Concomitantly, the primer at 900 nmol/l was tested for inhibition of the plasmid test system with 3 copies of the plasmid target in each well. Inhibition was judged to have occurred if the qPCR failed in all 3 replicates.
- the influence of the 3’ end of a primer on the production of nonspecific DNA amplicons and on inhibition was investigated by selecting sequences from within the *TCR*, *BCR*, *APC* and *GALT* genes such that a sequence had a Tm of 65–71°C, and, either the 2 most 3’ bases were G or C and the 2 most 5’ prime bases were A or T, or the 2 most 3’ bases were A or T and the 2 most 5’ prime bases were G or C. The sequence and its complement thus comprised a primer pair with the same Tm but with one member of the pair having 2 A or T bases at the 3’ end and the other member having 2 G or C bases at the 3’ end. A sequence and its complement were tested at 900 nmol/l for production of both nonspecificity and inhibition. Testing for nonspecificity used a gradient with annealing temperature ranging from 68–58°C and with 3 wells at each temperature. The mean Ct was determined from the wells at the 4 temperatures which ranged down from the highest temperature at which nonspecificity was evident in the 3 wells from both primers. Testing for inhibition used the plasmid test system with 3 copies of the plasmid target in each well. Inhibition was judged to have occurred if addition of the test primer at 900 nmol/l resulted in failure of the PCR in all 3 replicates.

### Ethics

Samples were obtained with written, informed consent from patients participating in study ALL06 of the Australasian Leukaemia and Lymphoma Group. The study was approved by the individual Ethics Committees of the hospitals at which the patients were treated and by the Southern Adelaide Clinical Human Research Ethics Committee (study 136.067). Patient data were fully anonymised.

## Results

**Fig 1:**
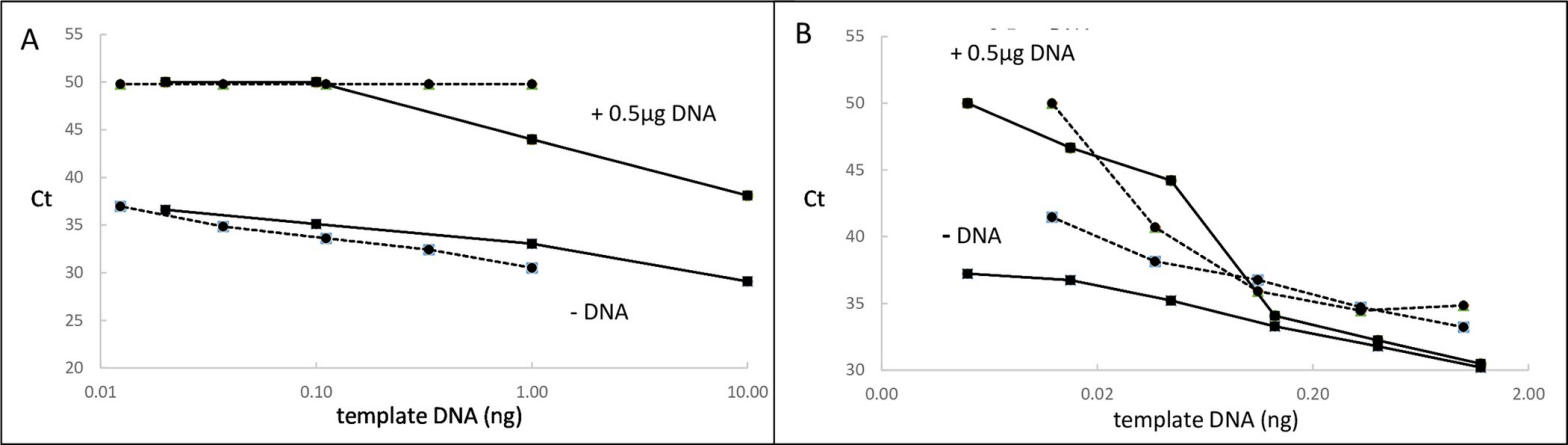
Inhibition of the PCR by genomic DNA. The primers were amplifying their target sequence in various masses of template DNA. An additional 0.5 μg of non-leukemic genomic DNA was or was not added to each well. In each panel, the 2 solid lines refer to one patient and the 2 broken lines refer to another patient. Panel A shows 2 examples of severe inhibition and panel B shows 2 examples of mild inhibition. A Ct of 50 indicates that the PCR failed and no signal was observed.

When non-target genomic DNA was not present in conventional qPCR, inhibition was generally not observed and amplification of a single copy of target DNA resulted in a Ct of 37–38. When genomic DNA, usually 0.5 μg, was present inhibition of the qPCR was seen with a substantial proportion of primers. Most commonly, as the mass of target DNA was progressively decreased, the qPCR was initially unaffected, then the Ct became unduly prolonged, then finally the qPCR failed completely. Table 1 in S1 Data shows an example of this effect. Fig 1 shows examples of different degrees of inhibition produced by 0.5 μg of genomic DNA. Panel A shows 2 examples of extreme inhibition in which prolongation of the Ct was already observed with masses of 1 or 10 ng of template DNA, masses which corresponded to MRD values of 2x10^-3^ and 2x10^-2^ respectively. Panel B shows 6 examples in which inhibition was only evident when the mass of template DNA was less than approximately 100 pg. This mass of template DNA in 0.5 μg genomic DNA is equivalent to an MRD level of 2x10^-4^.

**Fig 2:**
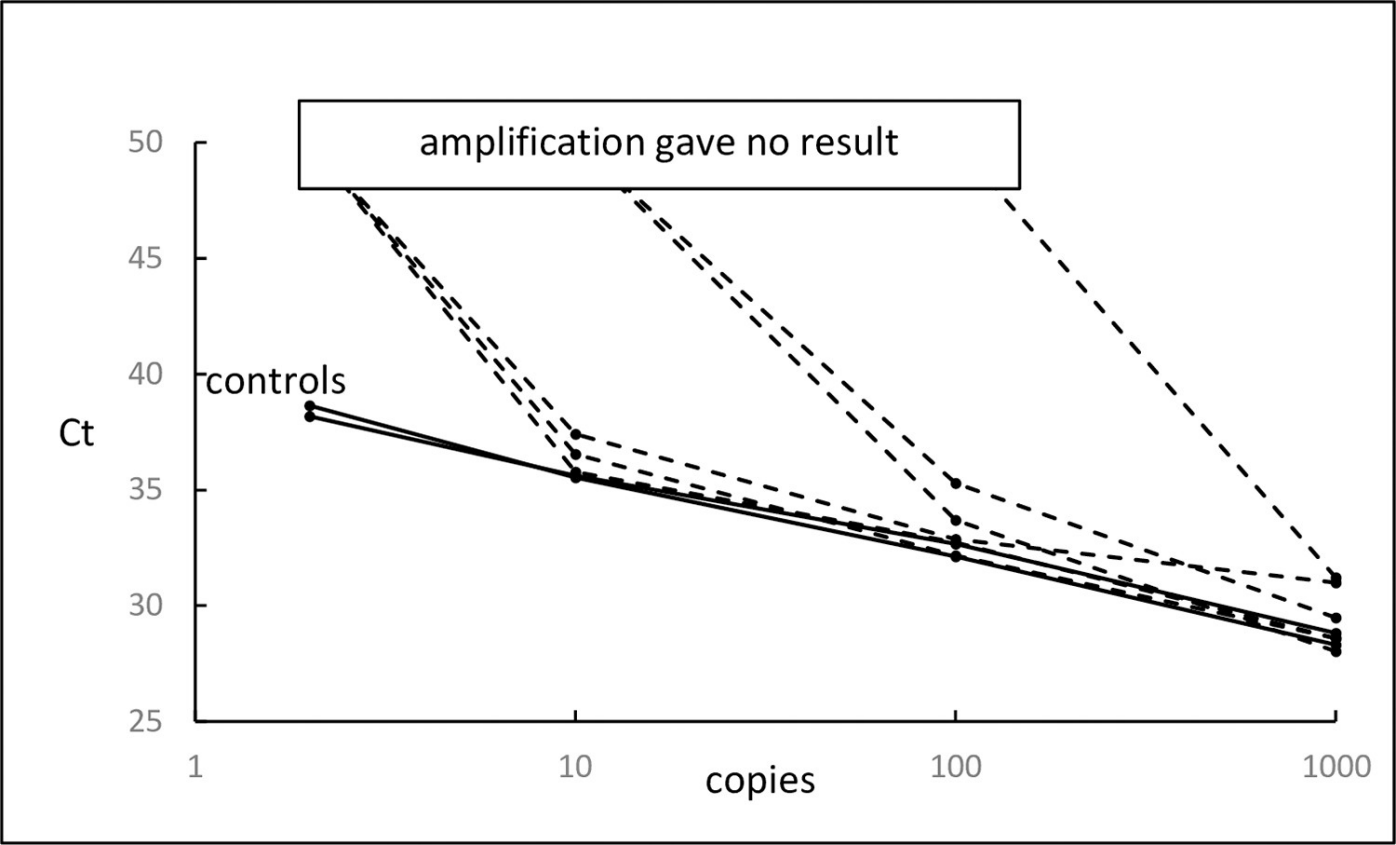
Effect of test primers on amplification of plasmid target in the presence of DNA. The effect of a primer pair from each of 6 patients on amplification of different numbers of plasmid sequences in the presence of 0.5 μg of genomic DNA was determined. Control reactions, which contained only the plasmid primers, are the solid lines, and the test reactions, which contained both the plasmid primers and a pair of patient primers, are the broken lines.

PCR inhibition was also seen when primer pairs were added to the plasmid test system, as shown in Fig 2 The number of plasmid copies in the reaction is shown and 100 copies in 0.5 μg genomic DNA is approximately equivalent to an MRD level of 10^−3^. Table 4 in S1 Data shows the reversal of inhibition of the plasmid system produced by increasing the annealing temperature.

**Fig 3:**
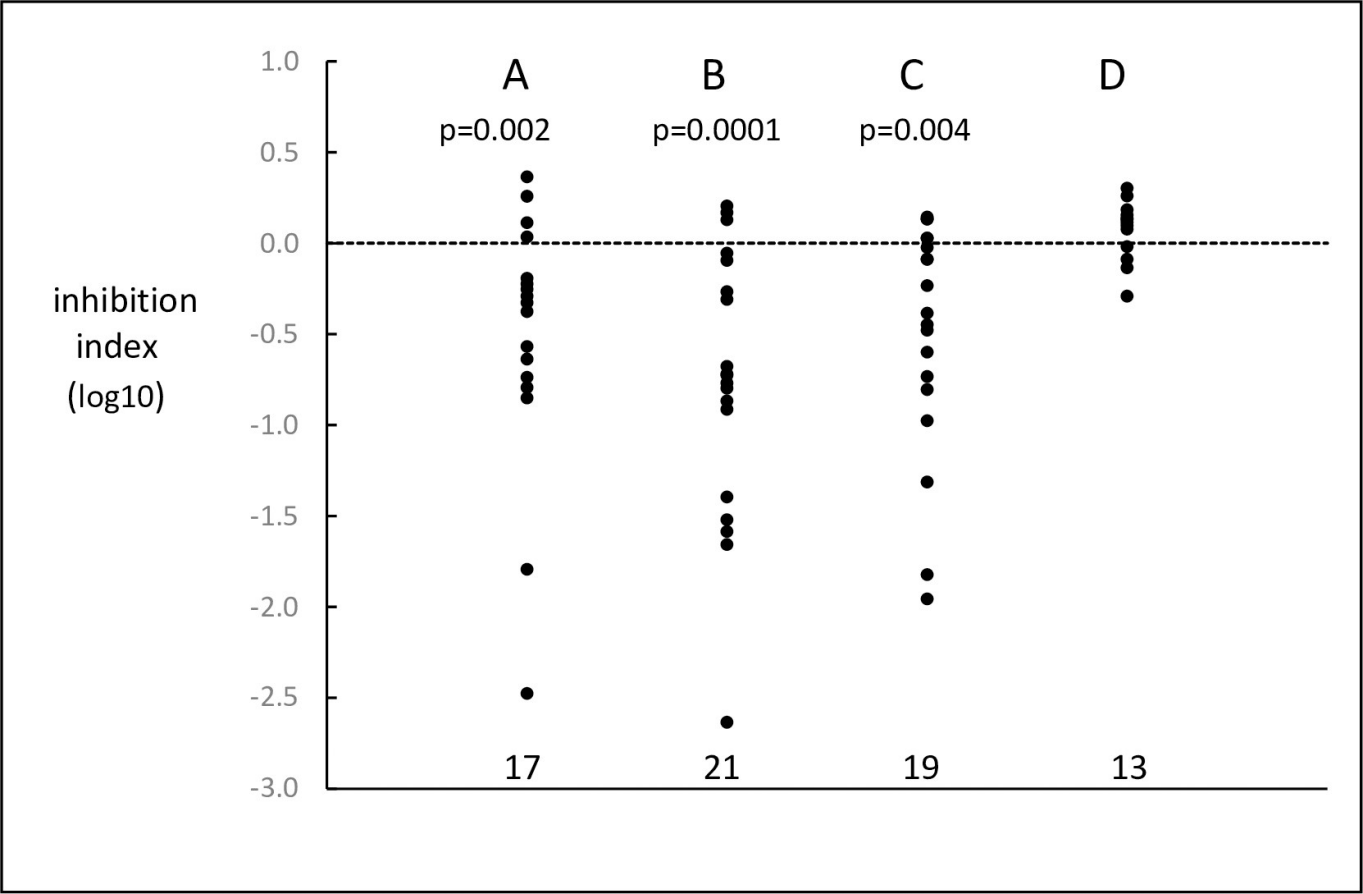
Inhibition indexes. Column A shows the results from amplification of patient DNA by patient primer pairs; Column B shows the results from amplification of plasmid DNA by plasmid primers in the presence of patient primer pairs; Column C shows the results from amplification of plasmid DNA by plasmid primers in the presence of widely recommended reverse primers; Column D shows the results from amplification of plasmid DNA by plasmid primers in the presence of HAT-PCR reverse primers and with amplification by the HAT-PCR protocol. The numbers of primer pairs or primers are shown towards the bottom of the Figure and at the top are shown the significance probabilities of the observed results, tested by the Wilcoxon matched-pairs signed ranks test against the null hypothesis of no inhibition.

The inhibition indexes observed with primer pairs or single primers are shown in Fig 3. Columns A, B and C show results obtained with conventional PCR. Column A shows the inhibitory effect observed when the patient primer pairs used for MRD assays were amplifying their specific genomic target; column B shows the inhibitory effect of patient primer pairs on amplification of the plasmid test system; column C shows the inhibitory effect on the plasmid system of the individual reverse J primers for *IGH and TCRβ* recommended by EuroMRD. The 3 most inhibitory primers were *IGH* J4, *TCRβ* J2.5 and *TCRβ* J2.7. Panel D shows that HAT-PCR J primers used under HAT-PCR conditions did not produce detectable inhibition. Comparison of Panel C with Panel D suggests that some degree of inhibition was produced by approximately half of the 19 EuroMRD primers. The inhibitory index of the EuroMRD primers was significantly and positively related to their G/C content (r = 0.64, p = 0.004) and Tm (r = 0.77, p < 0.001).

Table 1 shows the relationship between nonspecificity and inhibition by genomic DNA of the plasmid amplification system. Results are from 19 conventional reverse primers, and from 11 forward and 13 reverse HAT-PCR primers. Fishers exact test showed that the relationship between nonspecificity and inhibition was highly significant (p << 0.001) and that conventional primers produced significantly more nonspecificity (p = 0.008) and inhibition (p = 0.002) than did HAT-PCR primers.

**Table 1:** Frequency of nonspecificity and PCR inhibition produced by conventional PCR and HAT-PCR.

	nonspecificity	PCR inhibition		
		present	absent	
conventional PCR	present	7	0	19
	absent	0	12	
HAT-PCR	present	0	1	24
	absent	0	23	

Results were from a total of 43 primers.

Numerous factors influenced inhibition by genomic DNA. Inhibition decreased with decreasing concentration of DNA, decreasing concentration of magnesium, increasing annealing temperature and increasing concentration of polymerase. The effects of annealing temperature and Taq were seen with both target genomic DNA and the plasmid system. Table 2 shows an example of the results and Table 3 in S1 Data show additional results. Addition of albumen to the PCR up to 0.1% had no effect on inhibition.

**Table 2:** Effect of annealing temperature and Taq concentration on inhibition.

plasmid copies	test primers	Taq units	annealing temperature					
			66.7	65.1	63.2	61.6	60.6	60
2	**-**	0.6	38.11	37.23	38.08	38.13	37.71	38.16
2	pat_1_	0.6	37.27	40.47/2	N/A	N/A	N/A	N/A
2	pat_1_	2	36.22	38.45	41.53	40.22	40.28/1	39.21/2
2	pat_1_	4	37.34	36.76	39.20	37.40	37.24	37.69/2
2	**-**	0.6	37.24	35.62	35.24	36.15	35.93	37.21
2	pat_2_	0.6	N/A	N/A	N/A	N/A	N/A	N/A
2	pat_2_	2	37.39	38.62	37.13/2	N/A	N/A	N/A
2	pat_2_	4	38.04	36.46	37.68	36.63	39.26	39.15

The effect of addition of the primer pair from each of 2 patients on amplification of plasmid targets by plasmid primers was determined. Genomic DNA 500 ng was present in all reactions. There were 3 replicates for each point and the Ct value shown is the mean value of the positive amplifications. N/A indicates that no positive amplifications were observed. The additional number in several cells indicates the number of wells that showed positive amplification.

The magnitude of inhibition by EuroMRD J primers increased with increasing primer concentration, G/C content or Tm. Inhibition was greater when primer template rather than plasmid template was present in the reaction, which suggested that consumption of primers was a factor in the mechanism of inhibition. The necessity for primers to hybridise and extend in order to produce inhibition was shown by the observation that modification at their 3’ ends by either phosphorylation or addition of inverted dT, which blocked extension, prevented nonspecific amplification and inhibition of amplification of the plasmid sequence. The results for modified primers are shown in the Table 5 in S1 Data.

**Fig 4:**
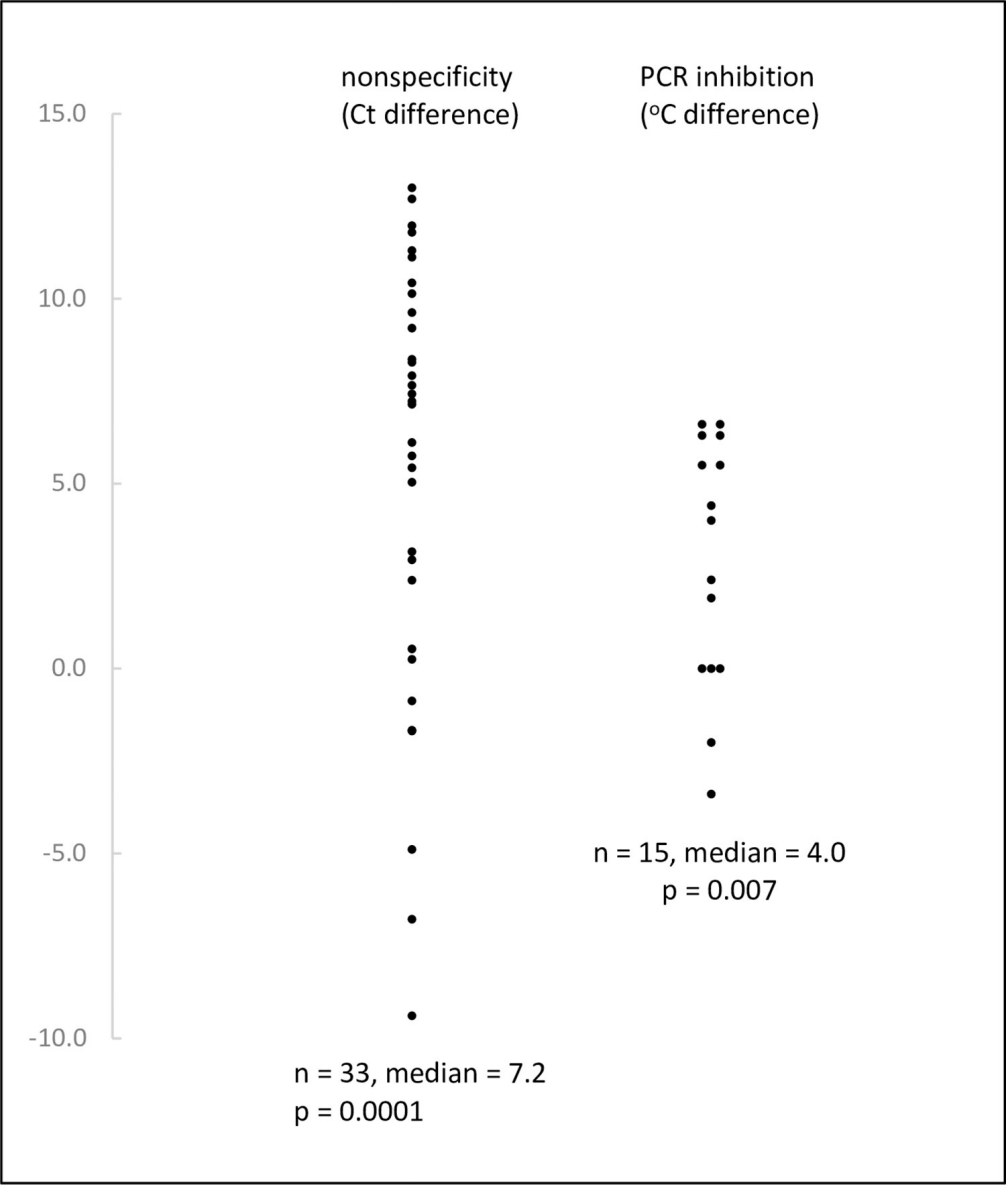
Effect of the 3’ end of the primer on production of nonspecificity and PCR inhibition. Experiments were performed with a gradient of annealing temperature. The Ct difference is the Ct for an A/T primer minus the Ct for a G/C primer and the positive value of the median indicated that, at the same annealing temperature, G/C primers evidenced a lower Ct and therefore more nonspecificity. The °C difference is the temperature difference between G/C and A/T primers in the minimum annealing temperature required to prevent inhibition. The positive value of the median indicated that G/C primers required a higher annealing temperature to prevent inhibition. The statistical test was the Wilcoxon matched-pairs signed-rank test.

Fig 4 shows the effect of the 3’ end of the primer on the production of nonspecificity and on inhibition of the plasmid sequence. Primers having a complementary sequence were compared to each other and primers ending in G or C produced significantly more nonspecificity and inhibition than primers ending in A or T.

## Discussion

**Fig 5:**
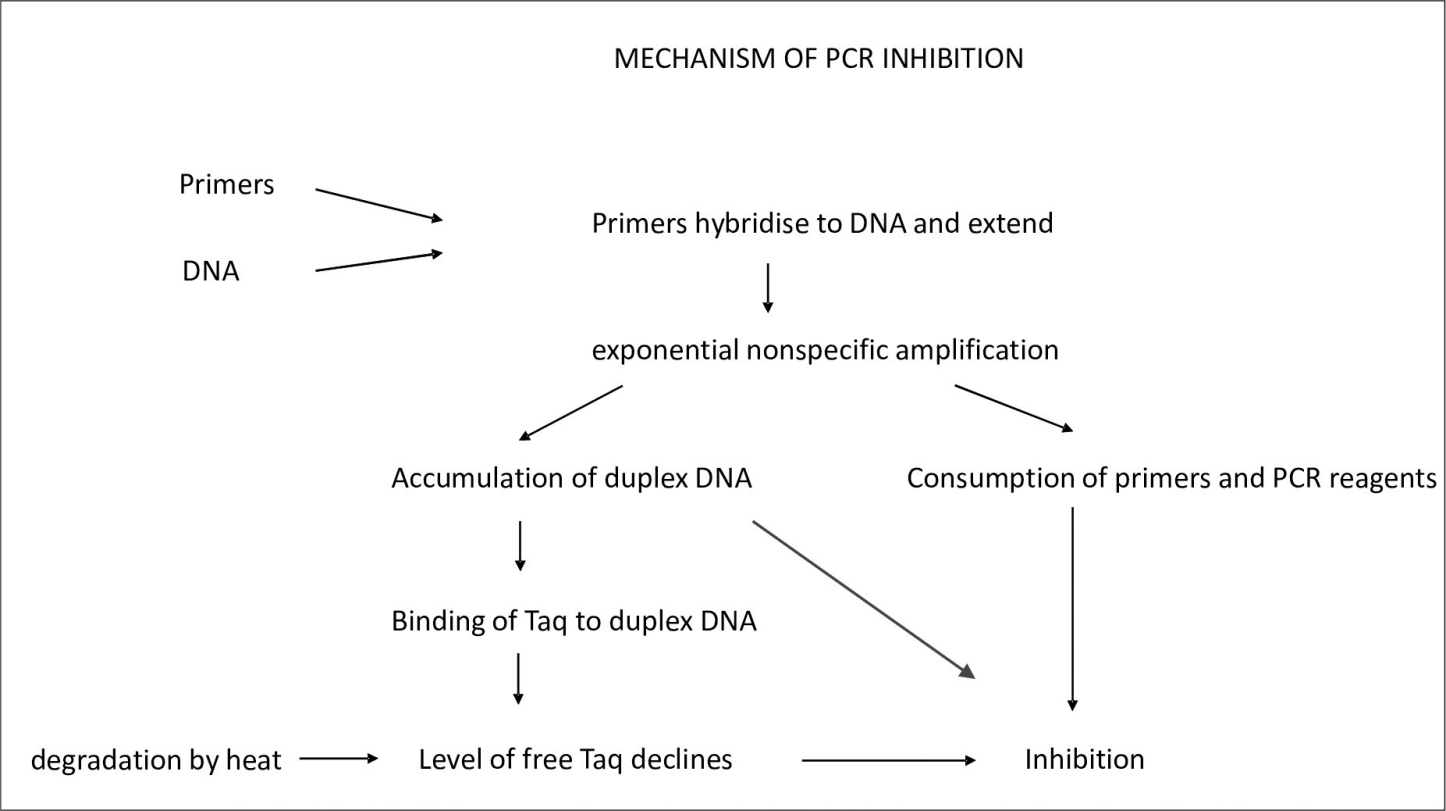
Proposed mechanism of inhibition of the PCR by genomic DNA.

Inhibition, as manifest by the plateau, always occurs in the PCR due to a combination of reagent consumption and end-product inhibition but in a successful PCR the desired endpoint is reached before inhibition becomes pronounced. Fig 5 shows our working hypothesis on the role of genomic DNA in production of inhibition. Non-specific hybridisation of a primer to DNA is favoured by a high G/C content and an annealing temperature which is low relative to primer Tm, and subsequent extension is facilitated by G/C bases at the 3’ end. Cross-hybridisation of non-specific amplicons results in exponential amplification of non-specific material. This consumes reagents and the increasing amount of double-stranded DNA binds Taq polymerase [12]. Owing to the exponentially increasing nature of the underlying mechanism, PCR inhibition by DNA may not be substantial until a large number of cycles, usually more than 30, has occurred and it may therefore only be manifest when a small number of target templates is being amplified.

Some previous PCR reports relate to our findings. Annealing temperature at the start of the PCR prevents primer-dimer formation [7]. Mecklenburg used a high annealing temperature throughout the PCR and separately coined the acronym of HAT-PCR, although the reasons for his use were the prevention of primer-dimer formation and optimisation of a biphasic PCR protocol [13]. There are few references to his work, and we were unaware of it when we did this study. Dietrich noted that inhibition of the PCR produced by degraded DNA could be ameliorated by an increased concentration of Taq polymerase [10]. The effect of mismatches indicates the importance of the 3’ end of the primer for binding and extension [14].

Our findings are relevant both for the particular issue of measurement of MRD by PCR and for the general issue of PCR design. We found that performing PCR according to the widely used EuroMRD guidelines often failed to quantify MRD in the 10^−4^ to 10^−6^ range. Analysis of the factors responsible mainly used the recommended generic reverse J primers, which are not patient-specific, and suggested the following. The relationship between annealing temperature of the PCR and primer Tm is suboptimal, as the annealing temperature of 60°C, which is recommended for use except when false positive results are obtained in controls, is 5–10°C below the Tm of the reverse primers. In terms of the 3’ end of the primer, the guidelines recommend that the patient-specific forward primers have no more than 2 G/C bases in the 5 most 3’ bases but make no recommendations for the reverse primers, some of which terminate with 3 or even 4 G/C bases. The concentration of Taq polymerase is not considered and our findings suggest that the concentration in several commercial kits is suboptimal.

Our findings explain why some primers designed for MRD assays fail to achieve adequate sensitivity. They also explain the increased sensitivity evidenced by HAT-PCR, as it lacks the features of conventional PCR found to contribute to inhibition. The design features of HAT-PCR comprise: 1–6 A or T bases at the most 3’ positions of both the forward and the reverse primer; the annealing temperature being within 3°C of the highest annealing temperature which still gives efficient amplification; a relatively high concentration of Taq polymerase. For HAT-PCR in the present study: the annealing temperature was 72°C; the Tm of the patient-specific forward primers was 69.0–70.5; the Tm of the generic reverse J primers was 1–2°C greater; the 3’ end of both forward and reverse primers comprised 1–4 A/T bases; and a 25 μL reaction contained 2 units of Taq polymerase. In this study HAT-PCR evidenced only one instance of slight nonspecificity and no instances of inhibition.

HAT-PCR was designed to address the problem of false-positive results due to binding of primers to non-target rearranged *IGH* and *TCR* sequences. However, its design also corrects the factors in conventional PCR identified in the present study as leading to promiscuous primer binding and extension, nonspecific amplification and PCR inhibition. As a result, HAT-PCR can detect MRD at a level of 10^−6^ and this increased sensitivity materially increases the clinical utility of PCR for measurement of MRD.

Our findings are also relevant to the general issue of PCR design. Quantification by PCR is increasingly employed for purposes such as detection of pathogens or mutations, the latter particularly in oncology, and this may involve many cycles of amplification to detect only one or a few targets embedded in a large mass of non-target DNA. Current recommendations on design of the PCR have been directed principally towards achieving efficient amplification of the target of interest. They allow a wide range of annealing temperature and polymerase concentration. The common recommendation that G or C be used at the 3’ end of the primer appears to be based on theoretical considerations as we have been unable to find any supporting experimental evidence for it. Measures which minimise primer-dimer formation are generally used but our results indicate that, when a large mass of DNA is present and targets are rare, the PCR also needs to be made more stringent in order to improve specificity while still maintaining efficiency. This can be achieved by optimising primer design, PCR annealing temperature, and polymerase concentration. Furthermore, in addition to intact genomic DNA, inhibition of the PCR could conceivably be produced by other forms of DNA including cDNA or fragmented DNA, such as ancient DNA or DNA extracted from FFPE tissue. Nucleic acid amplification may also on occasion be performed by a method other than PCR. One such method is isothermal amplification, and it seems possible that, if the temperature is too low, primers may bind to non-target DNA and prevent sensitive detection. The findings of the present study in relation to improving specificity while maintaining efficiency of amplification may therefore be applicable to design of a variety of amplification assays of human or non-human tissue.

## Supporting information

S1 Data (XLSX)
